# Transition Readiness Into Adult Health Care in Children With Inflammatory Bowel Disease

**DOI:** 10.7759/cureus.46825

**Published:** 2023-10-11

**Authors:** Hadeel A. Alsufyani, Sondos Jar, Wehad S Jambi, Nuha Meer, Weam Bajunaid, Hadeel A Albaradei, Nadin A Alharbi, Haneen Aziz, Mahmoud Mosli, Omar I Saadah

**Affiliations:** 1 Medical Physiology, King Abdulaziz University Faculty of Medicine, Jeddah, SAU; 2 Faculty of Medicine, King Abdulaziz University Hospital, Jeddah, SAU; 3 Faculty of Medicine, King Abdulaziz University Faculty of Medicine, Jeddah, SAU; 4 Gastroenterology, King Abdulaziz University Faculty of Medicine, Jeddah, SAU; 5 Pediatrics/Gastroenterology, King Abdulaziz University Faculty of Medicine, Jeddah, SAU

**Keywords:** adult, adolescent, pediatric, care, transition, readiness, inflammatory bowel disease

## Abstract

Background and aim

Approximately 25% of inflammatory bowel disease (IBD) cases are diagnosed before the age of 18 years. Compared to adults, pediatric IBD is more aggressive and progresses rapidly. It is important to have a well-structured transition process in place when patients are transferred from pediatric to adult care. We aimed to evaluate the readiness of Saudi adolescents with IBD to be transitioned from pediatric to adult care using the Transition Readiness Assessment Questionnaire (TRAQ).

Materials and methods

This cross-sectional study was carried out at King Abdulaziz University Hospital (KAUH), Jeddah, Saudi Arabia, between January and December 2021. Pediatric patients aged between 12-18 with confirmed IBD were recruited. The mean TRAQ component and the overall scores were calculated and analyzed.

Results

A total of 54 patients with IBD were included. The overall mean TRAQ scores were moderately high (3.60±0.78), including high mean values for individual domains of the TRAQ. In terms of components of TRAQ, no significant differences between males and females were encountered; however, there was a trend for males having higher scores than females in tracking health issues (P=0.07). Patients older than 15 years had higher overall scores than younger patients (P=0.04). The level of child education was found to be the only independent variable that correlated with higher overall scores (P=0.005).

Conclusions

In this cohort of Saudi adolescents with IBD, patients showed moderately high overall mean TRAQ scores reflecting high readiness for transitioning. While males demonstrated a trend for higher scores compared to females in tracking health issues, patients older than 15 had higher total scores relative to younger patients. More studies are needed to examine the impact of better transition readiness on the long-term outcome of IBD.

## Introduction

Inflammatory bowel disease (IBD) is a group of chronic disorders defined by a pathological defective immune response that causes prolonged inflammation of the gastrointestinal tract. It is classified into two subtypes, ulcerative colitis (UC) and Crohn’s disease (CD) [1]. Worldwide, the incidence of IBD has been increasing [2]. A study conducted between 2003 and 2012 demonstrated a significant increase in the incidence of pediatric IBD patients in Saudi Arabia [3].

According to a study that assessed changes in the incidence and prevalence of IBD worldwide, approximately 25% of IBD cases were diagnosed before the age of 18 years [4]. Compared to adults, IBD in pediatric patients tends to be more aggressive and progresses rapidly. Since the diagnosis of IBD carries significant morbidity and is lifelong, it is important to maintain ongoing care by successfully transitioning patients from pediatric to adult care [5].

The transition program is an educational healthcare intervention that focuses on training pediatric IBD patients to acquire self-management skills and to take on responsibilities before transmission to adult care [6]. Transferring IBD patients from pediatric to adult care is an extremely challenging and distressing process that can lead to poor disease control, increased risk of hospitalization, and loss of follow-up. Therefore, a well-organized systematic protocol needs to be implemented to ensure transition readiness, mental preparedness, and effective communication with medical staff [3].

The transition program aims to improve the quality of life for IBD patients, and this process needs adequate preparation to achieve rewarding outcomes; otherwise, the results could be disappointing. Due to the shortage of studies that assess the readiness of patients in Saudi Arabia, more research needs to be implemented to achieve a better outcome. Therefore, the purpose of this study was to evaluate the readiness of Saudi pediatric IBD patients to be transmitted to adult health care.

## Materials and methods

This cross-sectional study was conducted to assess the level of preparedness in the transition of pediatric patients with IBD into adult health care and to identify the ideal age for transition among these patients. This study was carried out at King Abdulaziz University Hospital (KAUH), Jeddah, Saudi Arabia, January-December 2021, and approved by the Research Biomedical Ethics Committee of KAUH (Reference No. 288-21). Pediatric patients of both sexes aged between 12-19 who were seen at the pediatric gastroenterology clinics with a confirmed diagnosis of IBD were recruited. Patients who were following up with another pediatric specialty, unable to answer the questionnaire due to stress or pain from an acute flare, patients with mental or learning disabilities, and patients/parents who refused consent were excluded. Medical records were reviewed, and information for each enrolled patient was obtained. Sociodemographic characteristics including gender, age, nationality, phone number of caregivers, and monthly income were recorded.

To evaluate the readiness of adolescents to be transitioned to adult care, interviews by phone or on-site in the pediatric gastroenterology clinics were conducted after obtaining the informed consent of each patient or their caregiver verbally. The Transition Readiness Assessment Questionnaire (TRAQ) is a patient-centered questionnaire that aims to evaluate the readiness of adolescents to be transitioned to adult care. It measures the ability of the patient to manage their health independently and recognize the gaps they need to bridge to have better outcomes in the transition [7]. TRAQ is one of the most widely used, validated, and reliable scales to assess transition readiness [7-8]. It consists of 20 questions covering five domains, including the ability of IBD pediatric patients to manage their medication, follow up on their appointments, track their health issues, build good relationships with their healthcare provider, and organize their daily activities. Each question is scored on a five-point scale (one to five), with a score of one being assigned for responses of "No, I do not know how" and a score of five for responses of "Yes, I always do this when I need to". Although no threshold score for transition readiness has been set, a higher score indicates more transition preparedness [9]. TRAQ has good internal reliability, and the overall scale of Cronbach's alpha was 0.930 (standardized 0.933) [7].

Data analysis was carried out using the Statistical Package for the Social Sciences, Version 27.0 (IBM Corp., Armonk, NY). The items of each component of the TRAQ were computed to give the average score according to the number of items. The distribution of continuous variables was tested using the Shapiro-Wilk test, which revealed that age, talking with providers, and managing daily activities were not normally distributed. Normally distributed variables were summarized using mean ± standard deviation (SD), while variables not normally distributed were summarized using median and interquartile range (IQR). Independent sample t-tests and Mann-Whitney tests were used to compare the means of two groups, while ANOVA and Kruskal-Wallis tests were used to compare the means of more than two groups, as appropriate. The significance level was determined to be P value<0.05).

## Results

Baseline characteristics

The study sample included 54 pediatric patients with IBD, N=37 (68.5%) had CD, and N=17 (31.5%) had UC. The median age was 16 years (range 11-19 years). Males represented N=29 (53.7%), and only N=7, (13%) were non-Saudis. Medications were used by 85.3% as a treatment. Fathers were the caregivers in half of the participants (N=27, 50%). More than half of the patients indicated high school education (N=29, 53.7%), and similarly, more than half of the parents completed education to a bachelor's degree level (N=32, 59.3%). The family income was average among N=38 (70.4.%), low among N=8 (14.8%,) and high among N=8 (14.8%) (Table 1). 

**Table 1:** Baseline characteristics of the study cohort of IBD patients IBD, Inflammatory bowel disease; CD, Crohn’s Disease; UC, Ulcerative Colitis

Variable	Category	Number	Percentage %
Age (years)	≤15	17	31.5%
	>15	37	68.5%
Nationality	Saudi	47	87%
	Non-Saudi	7	13%
Gender	Male	29	53.7%
	Females	25	46.3%
IBD* subtypes	CD**	37	68.5%
	UC***	17	31.5%
Caregivers	Mother	13	(24.1%)
	Father	27	50%
	Both	14	25.9%
Child education	Primary or below	2	(3.7%)
	Secondary	18	33.3%
	High school	29	53.7%
	Bachelor	5	9.3%
Parents education	Secondary or below	7	13%
	High school	9	16.7%
	Bachelor	32	59.3%
	Postgraduate degree	6	11.1%
Income	Low	8	14.8%
	Average	38	70.4%
	High	8	14.8%

Transition readiness assessment 

The items of each component of TRAQ were computed to produce a total score. The results showed that managing medications had a mean of 3.39±1.19, appointment keeping had a mean of 3.19±1.02, tracking health issues had a mean of 2.99±1.13, talking with a provider had a mean of 4.33±1.04, and managing daily activities had a mean of 4.09±1.02. The overall score had a mean of 3.60±0.78 (Table 2).

**Table 2:** Mean individual domain and overall TRAQ scores of adolescents with IBD TRAQ, Transition Readiness Assessment Questionnaire; IBD, Inflammatory bowel disease

TRAQ* Domains	Score (mean±SD)
Managing medications	3.39±1.19
Appointment keeping	3.19±1.02
Tracking health issues	2.99±1.13
Talking with providers	4.33±1.04
Managing daily activities	4.09±1.02
Overall TRAQ score	3.60±0.78

The components of TRAQ and the overall calculated scores were compared between males and females. No significant differences were found between males and females in the overall mean TRAQ scores and in each component, with only a trend for males having higher scores than females in the “tracking health issues” domain (P=0.07) (Table 3). 

**Table 3:** Mean individual domain and overall TRAQ scores of adolescents with IBD by gender TRAQ, Transition Readiness Assessment Questionnaire; IBD, Inflammatory bowel disease

Domain	Gender	Number	Mean score±SD	P value
Managing medications	Females	25	3.4±1.11	0.59
	Males	29	3.31±1,28	
Appointment keeping	Females	25	3.14±0.86	0.75
	Males	29	3.23±1.15	
Tracking health issues	Females	25	2.69±1.04	0.07
	Males	29	3.26±1.16	
Talking with providers	Females	25	4.24±1.08	0.55
	Males	29	4.41±1.02	
Managing daily activities	Females	25	3.93±1.07	0.27
	Males	29	4.24±0.98	
Overall TRAQ score	Females	25	3.49±0.69	0.37

Age was categorized into two groups (≤ 15 and > 15 years old). TRAQ scores were further tested for significant differences between the two age groups. The older age group showed statistically higher overall scores (P = 0.04). While no other specific components were statistically significant, the older age group had higher means (Table 4). 

**Table 4:** Mean individual domain and overall TRAQ scores of adolescents with IBD by age ​​​​​​​ * Statistical significance, P<0.05 TRAQ, Transition Readiness Assessment Questionnaire; IBD, Inflammatory bowel disease

Domain	Age category	Number	Mean score±SD	P value
Managing medications	≤15	17	2.99±1.26	0.09
	>15	37	3.58±1.14	
Appointment keeping	≤15	17	2.89±1.22	0.15
	>15	37	3.32±0.91	
Tracking health issues	≤15	17	2.91±1.08	0.72
	>15	37	3.03±1.17	
Talking with providers	≤15	17	3.94±1.09	0.06
	>15	37	4.51±0.98	
Managing daily activities	≤15	17	3.701±1.28	0.05
	>15	37	4.28±0.83	
Overall TRAQ score	≤15	17	3.29±0.92	0.04*
	>15	37	3.75±0.68	

Linear regression analysis of factors influencing the overall TRAQ score identified the level of child education as an independent variable for higher TRAQ score achievement (B coefficient = 0.726, P=0.005) (Table 5).

**Table 5:** Linear regression analysis of the average total TRAQ score in relation to multiple independent factors TRAQ, Transition Readiness Assessment Questionnaire; IBD, Inflammatory bowel disease; CI, Confidence Interval; Statistical significance, P<0.05

Variable	B coefficient	95% CI*		P value
		Lower	Upper	
Age category	-0.332	-1.077	0.414	0.38
Gender	0.190	-0.204	0.584	0.34
Caregiver	-0.059	-0.363	0.244	0.69
Level of child education	0.726	0.232	1.22	0.005**
Level of parental education	0.116	-0.133	0.364	0.354

## Discussion

The transition period from pediatric to adult care can be difficult for some patients with IBD. Preparing youth with IBD for the transition is a challenging clinical care issue. Pediatric gastroenterologists have limited guidance on optimizing preparation. Factors that have the most influence on the process that would provide a swift transition for patients remain unknown. Our study recruited 54 pediatric patients with IBD with a median age of 16 years (IQR: 11-19 years). The ratio of male to female patients recruited was 1.2:1. There is a higher reported prevalence of males diagnosed with IBD compared to females. Similarly, this has been reported by multiple studies suggesting a higher predominance of males reported with CD [10-14].

In addition, the components of TRAQ and overall calculated scores were compared between males and females. Male patients had comparatively higher mean values in all the components and the total TRAQ score, except for managing drugs, where females had a higher mean value. However, this difference was not statistically significant. Multiple studies report better readiness associated with the female gender [9,15-18]. Female patients show better preparation for the transition from pediatric to adult clinic. The literature does not corroborate with the findings of our study: although not significant, higher mean values were noted in TRAQ components and overall TRAQ score in male patients. Other studies that have utilized the TRAQ score for other chronic illnesses, like diabetes and rheumatic diseases in pediatrics, similarly reported higher TRAQ scores in female patients compared to male patients [19-20]. These previous studies suggest that females demonstrated a greater transition readiness by being more confident in their self-management and self-advocacy skills relative to males. Other studies that used TRAQ to measure healthcare transition also reported a higher female TRAQ score compared to males suggesting a greater development in maturity and self-care abilities among female youth [21-22]. In contrast to the prevailing findings in most medical projects, our research suggests that there isn't a notable maternal bias in responsibility towards both sons and daughters, which is applicable to our setting. Culturally, fathers in Middle Eastern countries are responsible for accompanying their children to appointments as opposed to mothers. This is evident in our findings, as more than half of the participants' caregivers were their fathers. This responsibility of the fathers has been instilled in the same manner in their sons, as pediatric male patients, being more responsible towards themselves for greater involvement in their family's responsibilities in the future [23]. In addition to gender, several other factors can influence TRAQ scores, including age, socioeconomic status, educational level, psychosocial factors, and cognitive abilities. Moreover, individuals who have been diagnosed for a longer period may become increasingly acquainted with their disease and its management.

Of the cohort studied, 70.4% belonged to families of average income, while 14.8% were of low-income families, and 14.8% were of high-income families. A systematic analysis of patients from 195 countries reporting on the global burden of IBD showed that regions with a high sociodemographic index have a higher age-standardized prevalence rate compared to regions with a low sociodemographic index [24]. This shows the disparity in diagnosis, which can be associated with few diagnostic centers in addition to unaffordability and inaccessibility for the public. This can be related to our findings as lower-income families have decreased access to healthcare facilities and specialized centers. In addition, losing work time for family caregivers is not really feasible for low-income families [18].

In our study, age was categorized into two groups, above and below 15 years of age. The older age group showed statistically higher total TRAQ scores (P <0.05) (Table 4). Hence, our study demonstrated that age was associated with higher TRAQ scores. The relationship between age and transition readiness into adult health care is consistent with findings from other studies on the transition process for IBD patients [25-26]. In addition, the significant relationship between TRAQ score and level of child education as an independent variable is consistent with the age-related difference in which older children tend to have a higher TRAQ score and a higher level of education. The mean score for the overall TRAQ score for the older age group was 3.75 ± 0.68. However, Gray et al. proposed a mastery level of 90% of transition readiness skills which reflects an overall score of 4.5, suggesting that an increased transition readiness may minimize the retention of young adult patients in pediatric care and produce a large number of patients who are better prepared for adult care [25]. 

This study is among the first clinical-based assessment of transition readiness skills in pediatric patients with IBD. The study findings obtained from the readiness assessment will be beneficial to clinical services towards helping our patients meet our benchmark standard. In addition, the use of a well-validated measure of transition readiness in our study allows the application of the assessment in clinical settings. A principal limitation of the study was the absence of follow-up, as this was a cross-sectional study. A follow-up period would have allowed us to demonstrate an association between TRAQ scores and disease-specific outcomes. The homogeneity of the study should be noted as patients enrolled were similar in terms of age, children's level of education, the majority being in secondary and high school, parent level of education, the majority obtaining undergraduate degrees, and with average income. The similar demographic status of the patients enrolled may have affected the significance of the TRAQ components toward the readiness of the transition. On the other hand, the homogeneity is also a strength of the study as it eliminates possible confounding variables. Finally, the TRAQ data were collected from an online survey based on adolescents' self-reporting of skills and knowledge and did not include any objective measures that confirmed that the adolescent achieved these skills. This could also have influenced findings, given the wide range of ages and different developmental stages within the adolescent study participants. 

## Conclusions

Results from this study show moderately high overall mean TRAQ scores, including high mean values for individual domains of the transition readiness assessment. Males have a trend for higher TRAQ scores compared to females in tracking health issues. Patients below the age of 15 years exhibit low TRAQ scores, indicating that they are not adequately prepared for the transition to adult clinics.
